# Influence of Methylprednisolone Pulse Therapy on Liver Function in Patients with Graves' Orbitopathy

**DOI:** 10.1155/2018/1978590

**Published:** 2018-10-21

**Authors:** Piotr Miśkiewicz, Anna Jankowska, Kinga Brodzińska, Justyna Milczarek-Banach, Urszula Ambroziak

**Affiliations:** Department of Internal Medicine and Endocrinology, Medical University of Warsaw, Banacha 1a, 02-097 Warsaw, Poland

## Abstract

**Purpose:**

Intravenous methylprednisolone (IVMP) pulse therapy is the first-line treatment in active moderate-to-severe Graves' orbitopathy (GO) and dysthyroid optic neuropathy (DON). One of the adverse effects of this therapy is liver dysfunction that can be mild (ALT < 100 U/L), moderate (ALT: 100–300 U/L), and severe defined as acute liver injury (ALI) (ALT > 300 U/L). ALI can be irreversible and fatal. The aim of the study was to evaluate the influence of two different schemes of therapy with IVMP in moderate-to-severe GO and DON on biochemical liver parameters.

**Materials and Methods:**

49 patients with moderate-to-severe GO were treated with IVMP in every week schedule (cumulative dose 4.5 g), and 19 patients with DON received 3.0 g IVMP (1.0 g/day for 3 consecutive days). AST, ALT, and total bilirubin were measured before treatment and after IVMP in the following selected pulses: after 0.5 g (A1), 3.0 g (A2), and 4.5 g (A3) in the group with moderate-to-severe GO and after 3.0 g IVMP in the group with DON (B1).

**Results:**

We observed a statistically higher level of AST and ALT after therapy with 3.0 g of IVMP (B1) than after 0.5 g (A1), 3.0 g (A2), and 4.5 g of IVMP (A3). Mild elevation of ALT was found in 4% and 11% of patients with moderate-to-severe GO and DON, respectively. Moderate elevation of ALT was found in 0% and 21% of patients with moderate-to-severe GO and DON, respectively. There were no cases of ALI.

**Conclusion:**

Therapy of GO with higher doses (1.0 g) of IVMP in consecutive days is associated with higher risk of liver damage than treatment with moderate doses (≤0.5 g) in every week schedule. This trial is registered with NCT03667157.

## 1. Introduction

Graves' orbitopathy (GO) is a disorder characterized by orbital soft tissue inflammation and oedema associated with glycosaminoglycan deposition and fibrosis [1]. The most frequent cause is Graves' disease, but it can also occur in Hashimoto's thyroiditis [2]. The classification is comprised based on the severity of orbital changes ranging from mild GO, moderate-to-severe GO, to sight-threatening GO, which includes dysthyroid optic neuropathy (DON) [3]. Untreated GO can result in the significant impairment of quality of life (QoL) [4]. Intravenous methylprednisolone (IVMP) pulse therapy is the first-line treatment in the active-phase of moderate-to-severe GO and DON [3]. This therapy is more effective and better tolerated than oral glucocorticoids (GCs) [5–12]. The current recommendation of the European Group of Graves' Orbitopathy (EUGOGO) is that cumulative doses of intravenous GCs should not exceed 8.0 g in each treatment course, and pulses should not be given on consecutive or alternate days, except in the case of DON [3]. This limitation is due to the necessity of the prevention of severe side effects that are rare but may be fatal. One of the most severe adverse events is acute liver injury (ALI), in some cases irreversible and/or fatal [12–14]. In four independent studies, the estimated morbidity and mortality of ALI was found to be 1–4% and 0.01–0.3%, respectively [15–18]. Since 2000, there were 5 reported fatal cases [16, 17, 19]. This study was performed to evaluate the influence of different schemes of therapy with IVMP in patients with moderate-to-severe GO and DON on biochemical liver parameters.

## 2. Material and Methods

### 2.1. Patients

We enrolled 68 patients (46 women and 22 men) to the study. Patients were admitted to the Department of Endocrinology at the Medical University of Warsaw for IVMP pulse therapy between 2012 and 2016. The inclusion criteria consisted of active, moderate-to-severe GO or DON; age ≥ 18 years; euthyroidism for at least 1 month (patients with hyperthyroidism treated with antithyroid drugs, after radiotherapy/surgical treatment on levothyroxin therapy if necessary, with euthyroid GO, or with Hashimoto's disease on levothyroxin therapy); and completion of 12 pulses in patients with moderate-to-severe GO and 3.0 g cycles of pulses in patients with DON. The exclusion criteria were alanine aminotransferase (ALT) and/or aspartate aminotransferase (AST) >2x upper limit of normal; active viral hepatitis; abdominal ultrasound examination indicating chronic changes in liver structure, including cirrhosis; present or past medical history of autoimmune hepatitis; previous GCs therapy within the last 6 months; alcohol abuse; active inflammation; and active neoplastic disease.

Depending on the severity according to EUGOGO recommendations, patients were divided into the following two groups: the first group with active, moderate-to-severe GO (49 patients) and the second group with DON (19 patients). Moderate-to-severe GO was diagnosed according to EUGOGO recommendations [3]. Diagnosis of DON in patients with GO was based on at least two signs, including (i) deterioration of VA (<1.0), (ii) loss of colour vision (more than two errors in Ishihara plates), (iii) optic disc swelling, and/or (iv) signs of DON in a magnetic resonance (MR) scan (presence of apical crowding and/or optic nerve stretching). Group characteristics are described in Table 1.

### 2.2. Study Design and Outcome Analysis

Laboratory tests were performed before treatment in all patients from both evaluated groups. Serum markers of exposure to HBV and HCV were checked: hepatitis B surface antigen (HBs-Ag), hepatitis B surface antibody (HBs-Ab), hepatitis B core antibody (HBc-Ab), and hepatitis C antibody (HCV-Ab). Serum autoantibodies associated with autoimmune hepatitis including anti-nuclear antibodies (ANA1), anti-smooth muscle antibodies (ASMA), anti-mitochondrial antibodies (AMA), and anti-liver kidney-microsomal antibodies (anti-LKM) were also assessed. Thyroid evaluation included measurement of: thyroid-stimulating hormone (TSH), free triiodothyronine (fT3), free thyroxine (fT4), and serum antithyroid autoantibodies including anti-thyroid peroxidase (aTPO), thyroglobulin antibodies (aTG), thyroid-binding inhibitory immunoglobulin (TBII).

**Figure 1:**
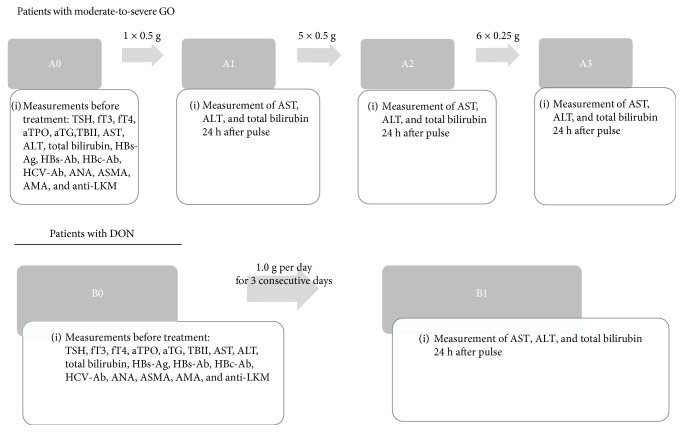
Treatment scheme. A0—patients with moderate-to-severe GO before treatment; A1—after administration of 0.5 g intravenous methylprednisolone (IVMP); A2—after administration of 3.0 g IVMP; A3—after administration of 4.5 g IVMP; B0—patients with DON before treatment; B1—after administration of 3.0 g IVMP; TSH—thyroid-stimulating hormone; fT3—free triiodothyronine; fT4—free thyroxine; aTPO—anti-thyroid peroxidase; aTG—thyroglobulin antibodies; TBII—thyroid-binding inhibitory immunoglobulin; AST—aspartate aminotransferase; ALT—alanine aminotransferase; HBs-Ag—hepatitis B surface antigen; HBs-Ab—hepatitis B surface antibody; HBc-Ab—hepatitis B core antibody; HCV-Ab—hepatitis C antibody; ANA—anti-nuclear antibodies; ASMA—anti-smooth muscle antibodies; AMA—anti-mitochondrial antibodies; anti-LKM—anti-liver kidney-microsomal antibodies; GO—Graves' orbitopathy; DON—dysthyroid optic neuropathy.

According to EUGOGO recommendations patients with moderate-to-severe GO were treated with IVMP cumulative dose 4.5 g during a 12-week period [3]. For the first 6 weeks 0.5 g IVMP per week was administrated and for the next 6 weeks 0.25 g IVMP per week. For the next 3 months patients received oral prednisone at a gradually reduced daily morning dose from 30 mg/day to 5 mg/day. Patients with DON received 3.0 g IVMP (1.0 g/day for 3 consecutive days) as the basic treatment [3]. Further treatment was established individually (endoscopic intranasal orbital decompression of medial wall, additional high doses of IVMP pulse therapy (3.0 g), additional orbital decompression (lateral, inferior or medial wall), 12 pulses with IVMP (additional cumulative dose 4.5 g)), but our statistical analysis included only an evaluation of the basic schedule. The effectiveness of such DON treatment was described in previous study by Miśkiewicz et al. [20]. Schemes of treatment and points of laboratory evaluation in both groups are presented in Figure 1.

Liver function parameters for further analysis including AST, ALT and total bilirubin were measured: the day before treatment (A0 in moderate-to-severe GO, B0 in DON) and the day after administration of IVMP in selected pulses: after 0.5 g (1st pulse—A1), after 3.0 g (6th pulse—A2) and after 4.5 g (12th pulse—A3) in the group with moderate-to-severe GO; after 3.0 g IVMP in the group with DON (B1). The comparison between groups, based on biochemical parameters, was performed in 3 stages: (i) A1 (0.5 g) *vs*. B1 (3.0 g), (ii) A2 (3.0 g) *vs*. B1 (3.0 g), and A3 (4.5 g) *vs*. B1 (3.0 g).

Depending on concentrations of ALT, liver dysfunction was divided into mild (above the upper limit of normal but less than 100 U/L), moderate (100–300 U/L), and severe (>300 U/L). ALI was defined as an ALT concentration > 300 U/L. However, we also evaluated a 4-fold increase of ALT in comparison to the initial values.

Routine laboratory tests and clinical evaluation were performed before every single pulse. Laboratory tests consisted of ALT, AST, CRP, and urine analysis. In cases of moderate and severe increases in ALT (more than 100 U/L), therapy was stopped. In patients with DON and increased ALT (more than 100 U/L) after 3.0 g IVMP, we did not repeat another cycle of 3.0 g IVMP. However, if ALI did not occur (ALT < 300 U/L) and the level of ALT/AST normalized, we administered 12 pulses of IVMP in every week schedule (additional cumulative dose 4.5 g) in these patients with an active phase of GO.

Follow-up was performed in all of the patients and it included evaluation of AST, ALT, and total bilirubin between 1 and 3 months after completion of IVMP therapy.

The study was approved by the Bioethics Committee of the Medical University of Warsaw.

### 2.3. Laboratory Measurements

Venous blood was collected in the morning following a 12-hour fast. TSH, fT3, fT4, aTPO, aTG, TBII, HBs-Ag, HBs-Ab, HBc-Ab, and HCV-Ab were measured in serum using an electrochemiluminescence immunoassay (cobas 8000 System, Roche Diagnostics, Switzerland). Concentrations of AST and ALT were measured by the standard reaction of NADH oxidation, total bilirubin by calorimetric method (cobas 8000 System, Roche Diagnostics, Switzerland). The level of serum autoantibodies (ANA1, ASMA, AMA, and anti-LKM) was measured using an indirect immunofluorescence method (EUROIMMUN tests, Sprinter XL Analyzer, EUROIMMUN, Germany).

All methods were calibrated and controlled according to manufacturers' recommendations. Laboratory normal ranges are presented in Table 1.

### 2.4. Ultrasound

Liver ultrasound examination was performed in every patient before IVMP therapy.

### 2.5. Statistics

Statistical analysis was performed using STATISTICA software ver. 12.0. Continuous variables were demonstrated as mean ± standard deviation (SD) or median values. Categorical data were presented as numbers (*n*) or percentages (%). Changes between liver function parameters in selected time points of the study were compared using the Mann–Whitney *U* test and Fisher's 2 × 2 exact test. The statistical significance was established at <0.05 level. After Bonferroni's correction for the six main tested hypotheses, results with a *p* value < 0.0083 were considered significant.

## 3. Results

### 3.1. Evaluation before Intervention

In 2 patients from the group with moderate-to-severe GO with a past medical history of viral hepatitis B, mild increases in ALT were observed before therapy, which later normalized during IVMP treatment. In 2 patients with DON and a past medical history of viral hepatitis B, the level of aminotransferases was within the normal range before and during therapy. In all of these patients, active viral infection was excluded. In one patient with moderate-to-severe GO and a history of HAV infection, serum aminotransferase concentrations were within the normal range during the entire treatment. Two patients with moderate-to-severe GO were suspected of Gilbert's syndrome. Laboratory measurements indicated an increased total bilirubin value during the entire IVMP therapy, while AST and ALT concentrations were within the normal limits. Mean ALT, AST, and total bilirubin levels were comparable between groups with moderate-to-severe GO (A0) and DON (B0) (Table 1). Serum autoantibodies associated with autoimmune hepatitis (ANA1, ASMA, AMA, and anti-LKM) were negative in all patients before treatment. One patient in the DON group had diabetes type 2 treated with metformin. Glucose control was good in this patient, and HbA1C was normal. In 18 patients (13 with GO and 5 with DON), the ultrasound revealed liver steatosis. Only in one patient with liver steatosis and moderate-to-severe GO the level of ALT was mildly increased before treatment (68 U/L). This aminotransferase normalized during the IVMP treatment.

### 3.2. Evaluation after IVMP Treatment

**Figure 2:**
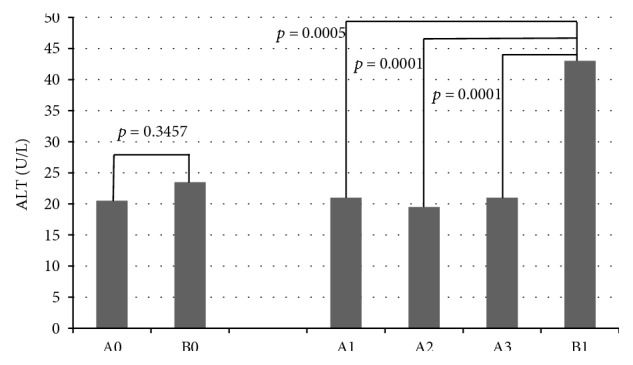
Comparison of the influence of different models of intravenous methylprednisolone IVMP therapy on ALT—median values and statistical significance using Mann–Whitney *U* test (*p* values after Bonferroni correction are significant if <0.0083). A0—before treatment; A1—after administration of 0.5 g IVMP; A2—after administration of 3.0 g IVMP; A3—after administration of 4.5 g IVMP; B0—before treatment; B1—after administration of 3.0 g IVMP; ALT—alanine aminotransferase (normal range: 7–56 U/L).

Analysed data are presented in Figure 2 and Tables 2 and 3. We observed a statistically higher level of AST and ALT after therapy with 3.0 g of IVMP (B1) in DON than in patients with moderate-to-severe GO after 0.5 g (A1), 3.0 g (A2), and 4.5 g (A3) of IVMP. Total bilirubin median values were similar in both groups of patients after therapy.

In patients with moderate-to-severe GO, a mild elevation of ALT was observed in 0 (0%), 2 (4%), and 0 (0%) patients in A1, A2, and A3 points of evaluation, respectively. There were no patients with moderate and severe increases of aminotransferases in this group.

In patients with DON in point B1, mild elevation of ALT was observed in 2 patients (11%) and moderate in 4 patients (21%). Severe increases of aminotransferases were not observed. The risk of moderate elevation of ALT in the DON group was higher than in moderate-to-severe GO (*p* = 0.0048). A 4-fold increase of ALT was observed after therapy in 4 patients with DON (21%) and in none of the patients with moderate-to-severe GO at all points of evaluation (A1, A2, and A3). These differences between both groups were statistically significant (*p* = 0.0048). Biochemical parameters did not progress to complete ALI and frankly normalized during follow-up. All patients with DON with increased ALT and/or AST were treated with additional 12 pulses of IVMP in every week schedule (additional cumulative dose 4.5 g) after previous normalization of aminotransferase levels. The levels of ALT and AST were in the normal range during this part of treatment (Table 3). We did not notice an increase of ALT after this therapy. Additionally, we did not observe moderate or severe increases of ALT and/or AST before routine pulses between the main points of evaluation (in both groups of patients).

Before and during IVMP therapy, 18 patients were treated with statins (11 patients with moderate-to-severe GO and 7 patients with DON). All of the patients had aminotransferases in the normal range before treatment. In 13 of the patients, aminotransferases were in the normal range during the entire IVMP therapy. In 4 patients after IVMP administration, there was a mild increase of ALT or AST observed (three patients with DON and one patient with moderate-to-severe GO). In one patient with DON, we found a moderate (>100 U/L), 4-fold increase of ALT.

There was no difference between the age of patients with DON with *vs.* without an increased level of ALT concentration 61.4 *vs.* 64.5 years (*p* = 0.83).

## 4. Discussion

IVMP pulse therapy is used as the first-line treatment in active moderate-to-severe and very severe GO [3]. It decreases inflammation and clinical symptoms and significantly improves the quality of life [4]. However, it is also associated with several side effects. One of these is hepatotoxicity, often manifested as an asymptomatic increase in serum liver enzymes. However, several cases of ALI due to IVMP have been reported, some of which were fatal [15–19, 21–26]. The morbidity and mortality of ALI were declared by several authors to range from 0.8% to 4.0% and from 0.09% to 0.3%, respectively (Table 4) [14–18, 27].

This is the first study to compare the influence on biochemical liver parameters of two treatment schedules with IVMP without oral CCs in the same evaluation. We observed that after 3.0 g IVMP given in three consecutive days (3 × 1.0 g), the increase of ALT was higher in comparison to therapy on a weekly schedule: 0.5 g (one pulse), 3.0 g (6 × 0.5 g), and 4.5 g (6 × 0.5 g plus 6 × 0.25 g). This indicates that higher single doses of IVMP (1.0 g) given on consecutive days are associated with a higher risk of hepatotoxicity than weekly pulses with medium or lower doses (≤0.5 g), even when the cumulative dose is lower (3.0 g *vs*. 4.5 g). To our knowledge, only one study, performed by Le Moli et al., compared the safety and efficacy of GCs therapy patients with active, moderate-to-severe GO and DON according to hepatotoxicity [28]. The group with moderate-to-severe GO (*n* = 14) was treated according to the same protocol as in our study (cumulative dose 4.5 g). Patients with DON (*n* = 13) received two 3.0 g cycles of pulses repeated in the second week followed by oral prednisolone (cumulative dose 8.45 g). Authors evaluated an increase of ALT above the upper limit (40 U/L) during therapy and follow-up and noticed it significantly more often in the DON group than in the GO group. No cases of severe ALI were observed. They concluded that mild elevation in liver enzymes is dose dependent. It is worth underlining that in the Le Moli study, comparison of the effect of total GCs between the two groups included prednisolone therapy and a follow-up period.

 Liver damage is defined as an increased level of ALT. This parameter is approved to be more specific for hepatocytes than AST, leading some authors to establish ALT concentration as a criterion of division of liver dysfunction into mild (<100 U/L), moderate (100–300 U/L), and severe (>300 U/L). ALI is defined as ALT concentration > 300 U/L [15, 26]. Some authors consider a 4-fold increase in ALT as an additional criterion of hepatotoxicity [14]. None of our 68 patients was observed to have severe or fatal ALI. However, a 4-fold increase of ALT, which is considered in some studies as ALI criterion, was observed in 21% of patients with DON after IVMP therapy and in none of the patients with moderate-to-severe GO (*p* = 0.0048). Biochemical parameters did not progress to complete ALI and normalized during follow-up. Some of the patients with DON after the first treatment cycle (1.0 g/day for 3 consecutive days) received 12 additional pulses of IVMP in every week schedule (cumulative dose 4.5 g). What is interesting is that even if they have increased ALT and/or AST after the first cycle, we did not observe again an increase of aminotransferases during treatment in the second cycle with lower doses of IVMP (second cycle was started after normalisation of aminotransferases). According to Sisti et al.'s retrospective studies, the risk of ALI morbidity accounts for about 1% [15, 16]. Significant correlation with ALI was confirmed for age and methylprednisolone cumulative dose, which were both greater in patients with ALI [16]. In our study, we did not find a difference in age between patients with DON with *vs.* without an increased level of ALT concentration. However, patients in the DON group were older than in the group with moderate-to-severe GO and values of biochemical parameters over the upper normal limit did occur at various ages (Tables 1–3).

Mechanisms causing an IVMP-induced toxic effect on hepatocytes remain incompletely elucidated. There are some possible hypotheses that may explain the occurrence of ALI. Firstly, GCs can lead to the reactivation of autoimmune hepatitis [12, 19, 21, 22]. Moleti et al. suggest two types of immune reaction—an immune “rebound phenomenon” following GCs withdrawal, which may trigger autoimmune hepatitis in those predisposed, and an immune-allergic or metabolic idiosyncratic reaction toward GCs [24, 26, 29]. In our study, serum autoantibodies associated with autoimmune hepatitis (ANA1, ASMA, AMA, and anti-LKM) were assessed in all patients before treatment and were negative. The second mechanism of ALI is the reactivation of viral hepatitis [17, 30, 31]. Marino et al. [17] and Le Moli et al. [28] pointed out that the presence of viral hepatitis markers might increase the relative risk of liver damage, and ALT concentration > 40 U/L is significantly associated with liver injury in such patients. However, Wichary and Gasińska concluded that relatively high doses of IVMP in patients with a past history of HBV infection were not a risk factor for the reactivation of HBV [30]. We excluded active hepatitis in all patients before therapy. No cases of reactivation of viral hepatitis after IVMP administration were observed. Finally, there is a well-known direct toxic effect of GCs on hepatocytes, probably dose dependent [28, 30, 32, 33]. Normalization in follow-up of previously increased levels of ALT after very high doses IVMP (3.0 g), without further increase after the next 12 weekly pulses, indicate a direct toxic and dose-dependent effect of IVMP that was transient and did not lead to ALI.

High doses and long-term use of GCs are associated with the development or exacerbation of nonalcoholic steatohepatitis [34]. Ultrasound evaluation performed before treatment with GCs revealed steatosis in 18 patients, and only in one patient with moderate-to-severe GO was the level of ALT mildly increased and normalised during IVMP treatment.

In our opinion, patients with DON and increased ALT (especially >100 U/L and/or more than 4-fold) after the first cycle with very high doses of IVMP (1.0 g/day for 3 consecutive days) should be treated in the second step of therapy with decompression. Further therapy with 12 weekly pulses of IVMP in these patients with active GO can be considered, after aminotransferase normalization, without a history of ALI and the presence of viral hepatitis.

## 5. Limitations

One limitation of this study was the relatively small number of patients. Five patients with an increase of aminotransferases were taking statins (4 with DON and 1 with moderate-to-severe GO). Sabini et al. found no correlation between using statins and occurrence of ALI in patients taking high doses of intravenous GCs [35], but we cannot exclude such influence as an additional risk factor.

## 6. Conclusion

The therapy of GO with higher doses (1.0 g) of IVMP on consecutive days is associated with a higher risk of liver damage than treatment with moderate doses (≤0.5 g) on a weekly schedule. This study supports EUGOGO's statement that the only indication for such therapy should be DON. Careful examination should be performed before initial therapy with IVMP including liver function parameters (ALT, AST, and total bilirubin), autoantibodies associated with autoimmune hepatitis, and exclusion of active hepatitis. Monitoring of ALT and AST should be performed before every pulse of IVMP because acute liver injury can be asymptomatic, and even mild or moderate elevations of biochemical parameters can progress to complete ALI.

## Figures and Tables

**Table 1:** Group characteristics.

	Moderate-to-severe GO	DON	*p* value
*Number of patients*	49	19	
*Etiology of Graves' ophthalmopathy*			
Graves' disease	48	19	0.5545
Hashimoto thyroiditis	1	0	0.7246
*Sex*			
Females	34	12	0.5011
Males	15	7	0.4610
*Age (years)*	52.4 ± 11.7	63.6 ± 11.3	0.0004
*Current smokers*	25	8	0.4439
*Past smokers*	37	15	0.5364
*Nonsmokers*	12	4	0.5413
*BMI (kg/m^2^)*	26.2 ± 4.4	25.6 ± 4.6	0.0844
*Median CAS*	4	3	0.7350
*^131^I radiotherapy*	10	5	0.4480
*Thyroidectomy*	5	2	0.6375
*Comorbidity*			
Liver steatosis	13	5	0.6207
*Statins*	11	7	0.1828
*Mean fT4 (pmol/L)*	16.44	19.79	0.2031
*Mean aTPO (U/mL)*	211	147	0.1044
*Mean aTG (U/mL)*	270	164	0.3672
*Mean TBII (IU/L)*	11	13	0.6657
*Mean AST (IU/L)*	21	23	0.1585
*Mean ALT (IU/L)*	25	26	0.3457
*Mean total bilirubin (μmol/l)*	11.12	11.97	0.5583

GO—Graves' orbitopathy; DON—dysthyroid optic neuropathy; BMI—body mass index; CAS—clinical activity score; TSH—thyroid-stimulating hormone (normal range: 0.27–4.2 IU/mL); fT3—free triiodothyronine (normal range: 3.1–6.8 pmol/L); fT4—free thyroxine (normal range: 12.0–22.0 pmol/L); aTPO—anti-thyroid peroxidase (normal range < 34 U/mL); aTG—thyroglobulin antibodies (normal range < 115 U/mL); TBII—thyroid-binding inhibitory immunoglobulin (normal range < 1.75 U/mL); AST—aspartate aminotransferase (normal range: 5–40 IU/L); ALT—alanine aminotransferase (normal range: 7–56 IU/L); total bilirubin (normal range: 3.42–20.52 *μ*mol/L).

**Table 2:** Range of parameters in the moderate-to-severe GO group with increased aminotransferases (values above the upper limit of normal are shown bold).

Moderate-to-severe GO group													
Patient	Age	ALT (7–56 U/L)				AST (5–40 U/L)				TB (3.42–20.52 *μ*mol/L)			
		A0	A1	A2	A3	A0	A1	A2	A3	A0	A1	A2	A3
1	42	**79**	34	20	18	35	20	7	10	16.07	6.84	6.67	6.67
2	38	**68**	56	51	37	28	22	25	18	4.10	5.47	8.38	6.50
3	43	26	40	28	33	21	14	9	14	**26.00**	**35.57**	**39.33**	**31.98**
4	55	20	21	21	17	19	18	16	19	**22.06**	17.10	18.81	20.52
5	73	18	16	18	14	22	17	16	14	**31.63**	**23.94**	**29.41**	**31.81**
6	45	18	17	19	25	22	20	12	20	**26.33**	7.35	13.51	17.44
7	54	28	28	29	42	31	25	29	**41**	4.45	8.72	6.33	14.54
8	46	53	51	**57**	23	25	23	16	20	12.65	10.77	12.48	7.18
9	44	12	39	**72**	14	13	17	20	8	13.85	9.58	15.73	12.83
10	24	12	16	15	37	17	13	11	21	18.81	17.10	**25.31**	19.67

ALT—alanine aminotransferase, AST—aspartate aminotransferase, TB—total bilirubin; A0—before treatment; A1—after administration of 0.5 g IVMP; A2—after administration of 3.0 g IVMP; A3—after administration of 4.5 g IVMP; GO—Graves' orbitopathy.

**Table 3:** Range of parameters in DON group with increased aminotransferases or total bilirubin level.

DON group																			
Patient	Age	ALT (7–56 U/L)						AST (5–40 U/L)						TB (3.42–20.52 *μ*mol/L)					
		B0	B1	C0	C1	C2	C3	B0	B1	C0	C1	C2	C3	B0	B1	C0	C1	C2	C3
1	76	38	55	21	23	25	31	34	**44**	25	21	21	24	**22.74**	16.07	8.89	9.75	11.80	17.10
2	65	45	43	20	22	29	18	28	17	20	19	14	12	**26.85**	16.07	20.18	17.96	**22.06**	16.42
3	31	24	**193** ^∗^	11	19	21	—	21	**104** ^∗^	18	26	21	—	**24.11**	**21.34**	15.56	16.07	**24.45**	—
4	74	18	**238** ^∗^	14	15	17	18	17	**94** ^∗^	15	13	12	12	**21.72**	11.29	20.18	19.67	13.68	7.87
5	69	16	**95** ^∗^	41	37	28	33	26	**57**	23	25	12	14	14.54	6.16	9.23	9.23	7.70	8.21
6	76	32	**66**	25	36	33	21	37	**76**	29	31	32	18	8.04	6.84	6.33	7.01	6.16	8.38
7	50	39	**112**	32	26	21	16	35	**43**	21	17	9	13	4.62	7.18	9.41	7.87	5.64	7.35
8	69	24	**103** ^∗^	22	25	17	19	12	**88** ^∗^	18	12	12	14	12.65	16.07	10.94	13.51	20.52	11.80

Values above the upper limit of normal are shown in bold. ^∗^4-Fold increase of aminotransferase concentrations in comparison to initial values. ALT—alanine aminotransferase; AST—aspartate aminotransferase; TB—total bilirubin; B0—before treatment; B1—after administration of 3.0 g IVMP; C0—before continuation of treatment with additional 12 pulses of IVMP in every week schedule (4.5 g IVMP); C1—after administration of additional 0.5 g IVMP; C2—after administration of additional 3.0 g IVMP; C3—after administration of additional 4.5 g IVMP; DON—dysthyroid optic neuropathy; IVMP—intravenous methylprednisolone.

**Table 4:** Studies that evaluated the influence of IVMP on liver function in patients with moderate-to-severe Graves' orbitopathy and DON.

Study	Scheme of treatment	Number of patients	ALI morbidity *N* (%)	ALI mortality *N* (%)	Comments
(1) Current study	GO: 6 × 0.5 g + 6 × 0.25 g of IVMP on a weekly schedule *vs.*	GO: 49	Mild: GO—2 (4%), DON—2 (11%)	GO: 0	ALI—ALT concentration > 300 U/L
			Moderate: GO—0, DON—4 (21%)		ALT increase—mild (40–100 U/L), moderate (100–300 U/L), severe (>300 U/L)
	DON: 3 × 1.0 g on consecutive days	DON: 19	Severe: ALI—0	DON: 0	
(2) Le Moli et al. [28]	GO: 6 × 0.5 g + 6 × 0.25 g of IVMP on a weekly schedule *vs.*	GO: 14	0	0	Lack of ALI criterion. Patients with AST and/or ALT above the upper limit—40 U/L. Comparison of the effect of total GCs between the two groups of patients including those with prednisolone therapy.
	DON: 3 × 1.0 g of IVMP on consecutive days in the first week, repeated during the second week, followed by oral prednisolone in a tapering dose; CD of steroids 8.45 g	DON: 13	0	0	
(3) Marino et al. [17]	GO: CD of IVMP 3.0–24 g	GO: 800	7 (0.8%)	3 (0.3%)	Lack of ALI criterion
(4) Wichary and Gasińska [30]	GO: CD of IVMP 8 g in 4 weeks	GO: 30	0	0	Lack of ALI criterion
(5) Bartalena et al. [14]	GO: CD of IVMP 2.25–7.47 g	GO: 159	0	0	ALI—4-fold increase of aminotransferases
(6) Curro et al. [27]	DON: CD of IVMP 3.0–10.5 g	DON: 24	0	0	ALI—4x the upper limit of normal
(7) Eguchi et al. [18]	GO and DON: 1 g/d of IVMP for 3 consecutive days/week, repeated for 3–6 cycles, followed by a tapering dose of oral prednisolone. The daily dose of MP was reduced to 0.5 g except in cases with DON	GO and DON: 175	Mild 62 (35%),	0	ALT increase—mild (40–100 U/L), moderate (100–300 U/L), severe (>300 U/L)
			Moderate 10 (6%),		
			Severe 7 (4%)		
(8) Sisti et al. [16]	GO 4 different treatment protocols; CD of IVMP 1.2–30 g	GO: 1076	14 (1.3%)	1 (0.09%)	ALI—ALT > 300 IU/L
(9) Sisti et al. [15]	GO 4 × 15 mg/kg for 4 weeks and 8 × 7.5 mg/kg for 8 weeks; CD 3.8–13.3 g	GO: 376	4 (1.06%)	0	ALI—ALT > 300 IU/L

GO—moderate-to-severe Graves' orbitopathy; DON—dysthyroid optic neuropathy; ALI—acute liver injury; IVMP—intravenous methylprednisolone; CD—cumulative doses; ALT—alanine aminotransferase; AST—aspartate aminotransferase.

## Data Availability

The data (laboratory measurements) used to support the findings of this study are available from the corresponding author upon request.

## References

[1] Bahn R. S. (2010). Graves’ ophthalmopathy. *The New England Journal of Medicine*.

[2] Bartalena L., Pinchera A., Marcocci C. (2000). Management of Graves’ ophthalmopathy: reality and perspectives. *Endocrine Reviews*.

[3] Bartalena L., Baldeschi L., Boboridis K. (2016). The 2016 European Thyroid Association/European Group on Graves’ Orbitopathy guidelines for the management of Graves’ orbitopathy. *European Thyroid Journal*.

[4] Wiersinga W. M., Wiersinga W. M., Kahaly G. J. (2010). Quality of life. *Graves’ Orbitopathy — A Multidisciplinary Approach, Questions and Answers*.

[5] Zang S., Ponto K. A., Kahaly G. J. (2011). Intravenous glucocorticoids for Graves’ orbitopathy: efficacy and morbidity. *The Journal of Clinical Endocrinology & Metabolism*.

[6] Marcocci C., Bartalena L., Tanda M. L. (2001). Comparison of the effectiveness and tolerability of intravenous or oral glucocorticoids associated with orbital radiotherapy in the management of severe Graves’ ophthalmopathy: results of a prospective, single-blind, randomized study. *The Journal of Clinical Endocrinology & Metabolism*.

[7] Aktaran Ş., Akarsu E., Erbağci İ., Araz M., Okumuş S., Kartal M. (2007). Comparison of intravenous methylprednisolone therapy vs. oral methylprednisolone therapy in patients with Graves’ ophthalmopathy. *International Journal of Clinical Practice*.

[8] Kahaly G. J., Pitz S., Hommel G., Dittmar M. (2005). Randomized, single blind trial of intravenous versus oral steroid monotherapy in Graves’ orbitopathy. *The Journal of Clinical Endocrinology & Metabolism*.

[9] Kauppinen-Makelin R., Karma A., Leinonen E. (2002). High dose intravenous methylprednisolone pulse therapy versus oral prednisone for thyroid-associated ophthalmopathy. *Acta Ophthalmologica Scandinavica*.

[10] Macchia P. E., Bagattini M., Lupoli G., Vitale M., Vitale G., Fenzi G. (2001). High-dose intravenous corticosteroid therapy for Graves’ ophthalmopathy. *Journal of Endocrinological Investigation*.

[11] Stiebel-Kalish H., Robenshtok E., Hasanreisoglu M., Ezrachi D., Shimon I., Leibovici L. (2009). Treatment modalities for Graves’ ophthalmopathy: systematic review and metaanalysis. *The Journal of Clinical Endocrinology & Metabolism*.

[12] Miśkiewicz P., Kryczka A., Ambroziak U. (2014). Is high dose intravenous methylprednisolone pulse therapy in patients with Graves’ orbitopathy safe?. *Endokrynologia Polska*.

[13] Marcocci C., Watt T., Altea M. A. (2012). Fatal and non-fatal adverse events of glucocorticoid therapy for Graves’ orbitopathy: a questionnaire survey among members of the European Thyroid Association. *European Journal of Endocrinology*.

[14] Bartalena L., Krassas G. E., Wiersinga W. (2012). Efficacy and safety of three different cumulative doses of intravenous methylprednisolone for moderate to severe and active Graves’ orbitopathy. *The Journal of Clinical Endocrinology & Metabolism*.

[15] Sisti E., Coco B., Menconi F. (2015). Intravenous glucocorticoid therapy for Graves’ ophthalmopathy and acute liver damage: an epidemiological study. *European Journal of Endocrinology*.

[16] Sisti E., Coco B., Menconi F. (2015). Age and dose are major risk factors for liver damage associated with intravenous glucocorticoid pulse therapy for Graves’ orbitopathy. *Thyroid*.

[17] Marino M., Morabito E., Brunetto M. R., Bartalena L., Pinchera A., Marocci C. (2004). Acute and severe liver damage associated with intravenous glucocorticoid pulse therapy in patients with Graves’ ophthalmopathy. *Thyroid*.

[18] Eguchi H., Tani J., Hirao S. (2015). Liver dysfunction associated with intravenous methylprednisolone pulse therapy in patients with Graves’ orbitopathy. *International Journal of Endocrinology*.

[19] Weissel M., Hauff W. (2000). Fatal liver failure after high-dose glucocorticoid pulse therapy in a patient with severe thyroid eye disease. *Thyroid*.

[20] Miśkiewicz P., Rutkowska B., Jabłońska A. (2016). Complete recovery of visual acuity as the main goal of treatment in patients with dysthyroid optic neuropathy. *Endokrynologia Polska*.

[21] Salvi M., Vannucchi G., Sbrozzi F. (2004). Onset of autoimmune hepatitis during intravenous steroid therapy for thyroid-associated ophthalmopathy in a patient with Hashimoto’s thyroiditis: case report. *Thyroid*.

[22] Marinò M., Morabito E., Altea M. A. (2005). Autoimmune hepatitis during intravenous glucocorticoid pulse therapy for Graves’ ophthalmopathy treated successfully with glucocorticoids themselves. *Journal of Endocrinological Investigation*.

[23] Rivero Fernández M., Riesco J. M., Moreira V. F. (2008). Recurrent acute liver toxicity of intravenous methylprednisolone. *Revista Espanola De Enfermedades Digestivas*.

[24] Gutkowski K., Chwist A., Hartleb M. (2011). Liver injury induced by high-dose methylprednisolone therapy: a case report and brief review of the literature. *Hepatitis Monthly*.

[25] Melamud B., Lurie Y., Goldin E., Levi I., Esayag Y. (2014). Methylprednisolone-induced liver injury: a diagnostic challenge. *Israel Medical Association Journal*.

[26] Moleti M., Giuffrida G., Sturniolo G. (2016). Acute liver damage following intravenous glucocorticoid treatment for Graves’ ophthalmopathy. *Endocrine*.

[27] Currò N., Covelli D., Vannucchi G. (2014). Therapeutic outcomes of high-dose intravenous steroids in the treatment of dysthyroid optic neuropathy. *Thyroid*.

[28] le Moli R., Baldeschi L., Saeed P., Regensburg N., Mourits M. P., Wiersinga W. M. (2007). Determinants of liver damage associated with intravenous methylprednisolone pulse therapy in Graves’ ophthalmopathy. *Thyroid*.

[29] Gunawan B. K., Kaplowitz N. (2007). Mechanisms of drug-induced liver disease. *Clinics in Liver Disease*.

[30] Wichary H., Gasińska T. (2012). Methylprednisolone and hepatotoxicity in Graves’ ophthalmopathy. *Thyroid*.

[31] Bahn R. (2012). High-dose intravenous glucocorticoid therapy for Graves’ ophthalmopathy: where are we now?. *Thyroid*.

[32] Kaplowitz N. (2004). Drug-induced liver injury. *Clinical Infectious Diseases*.

[33] Dourakis S. P., Sevastianos V. A., Kaliopi P. (2002). Acute severe steatohepatitis related to prednisolone therapy. *The American Journal of Gastroenterology*.

[34] Farrell G. C. (2002). Drugs and steatohepatitis. *Seminars in Liver Disease*.

[35] Sabini E., Sisti E., Coco B. (2016). Statins are not a risk factor for liver damage associated with intravenous glucocorticoid pulse therapy for Graves’ orbitopathy. *Journal of Endocrinological Investigation*.

